# The capacity of schizophrenia and bipolar disorder individuals to make autonomous decisions about pharmacological treatments for their illness in real life: A scoping review

**DOI:** 10.1002/hsr2.179

**Published:** 2020-08-09

**Authors:** Enric Vincens Pons, Luis Salvador‐Carulla, Alfredo Calcedo‐Barba, Silvia Paz, Thomas Messer, Bruno Paccardi, Scott L. Zeller

**Affiliations:** ^1^ Department of Psychiatry Parc Sanitari Sant Joan de Déu, Sant Boi de Llobregat Barcelona Spain; ^2^ Centre for Mental Health Research Research School of Population Health, College of Health and Medicine, Australian National University Canberra Australia; ^3^ Department of Psychiatry, Hospital Gregorio Marañón Medical School at the Universidad Complutense de Madrid Madrid Spain; ^4^ SmartWriting4U Benicassim Spain; ^5^ Department of Psychiatry Danuviusklinik Pfaffenhofen Germany; ^6^ Psychiatric Unit Santa Chiara University Hospital, University of Pisa Pisa Italy; ^7^ Department of Psychiatry University of California California USA

**Keywords:** autonomy, bipolar disorder, capacity, schizophrenia, scoping review, treatment decision making

## Abstract

**Background and aim:**

Having decision making capacity is central to the exercise of autonomy in mental health care. The objective of this scoping review is to summarize the evidence on the capacity of people with schizophrenia or bipolar disorder to make decisions about their treatment in real life to support medical practice.

**Methods:**

Systematic search of observational studies on the assessment of capacity of patients with schizophrenia, psychosis, or bipolar disorder to make healthcare and treatment‐related decisions, conducted in any clinical setting published up to January 31, 2020 was performed. Free text searches and medical subject headings in English were combined in PubMed, Scopus, CINAHL, and PsycInfo. Publications were selected as per inclusion and exclusion criteria. The Newcastle‐Ottawa Scale for observational studies was used to assess the quality of publications.

**Results:**

Thirty publications were reviewed. According to the Newcastle‐Ottawa Scale criteria, the publications reviewed were good quality. Findings showed that more than 70% of schizophrenia and schizoaffective disorder outpatients understood treatment options at the point of making decisions about their illness and healthcare. Patients treated voluntarily had considerably better scores for decisional capacity than those treated involuntarily. The burden of psychiatric symptoms could compromise decisional capacity temporarily. Decision‐making capacity improved over time from admission to discharge from hospital, and with treatment among psychiatry inpatients. Schizophrenia and bipolar disorder patients could be as competent as nonpsychiatric individuals in making decisions about their treatments in everyday life.

**Conclusions:**

This scoping review provides a body of evidence for healthcare professionals in need of assessing the capacity of schizophrenia and bipolar disorder patients for autonomously decide about their treatments. Decisional capacity judgements should consider variations in capacity over time and be based on the type of decision to be made, the severity of symptoms, and the specific phase of the mental disorder.

## 1 INTRODUCTION

Respect for autonomy is a key principle in biomedical ethics. It is, however, a particularly vulnerable principle in everyday mental health care practice.^1^ In medical practice, autonomy is usually expressed as the right of competent adults to make informed decisions about their own medical care; it denotes self‐government.^2^ Autonomy is strongly associated with the idea that patients should be allowed and enabled to make their own decisions about treatments they receive for their diseases, and to make these decisions with purpose, substantial understanding, and freedom from controlling influences.^3^

A range of factors such as interactions with health‐care providers and symptom management challenge patient autonomy across a variety of diseases.^4^, ^5^ Preserving independence and privacy and dealing successfully with threats to self‐identity may enhance patients' autonomous decision making. Policies across the world require that, where possible, equal weight be given to the wishes, feelings, beliefs, and values of patients who have decision‐making capacity and of patients who are deemed to lack it.^6^ Psychiatry has led major improvements in patient empowerment as part of the development of person‐centered care and recovery.^7^ However, paternalistic attitudes could prevail despite an awareness of patients' right to autonomy and the practitioners' duty of reciprocity that requires to build up trust with the patient and to involve him or her in the planning and implementation of care.^8^

It is important to distinguish between the concepts of capacity and competency. Capacity describes a person's ability to a make a particular decision, whereas competency is a global assessment and a legal determination made by a judge in court.^9^ In a medical context, capacity refers to the ability to use information about an illness and proposed treatment options to make a choice that is congruent with one's own values and preferences^10^; it is the determining element that establishes the role of patient choices in medical decisions. Historically, patients with severe mental illnesses have been regarded as having impaired capacity for making functional decisions with respect to their health, and their agency has been largely disregarded in diagnosis and management.^11^ Persons with schizophrenia, for instance, describe a sense of being considered incapable and unmotivated to exercise their autonomy by their care givers, despite the knowledge that being trusted in their abilities and being offered freedom to make their own decisions might help them to respond successfully to a series of situations in daily life.^12^ Among bipolar disorder patients, medication schedules and a better understanding of illness and of treatment complications would foster better treatment decision‐making and adherence.^13^ In both cases, the assessment of capacity is critical for the agreement with the therapists in shared contracts^14^ and with the case‐manager in joint care planning.^15^

Assessing individuals' capacity to consent to or refuse treatment is a demanding task for psychiatrists, psychologists, and other healthcare professionals, particularly when dealing with unrepresented patients.^16^ It requires the assessment of the individual's ability to understand their medical situation and its consequences, to form and communicate a choice about the proposed care options, and to process the information in order to reach a rational decision. The evaluation may happen in episodes of acute care during a crisis and it must find a balance between promoting and restoring the patient's health, providing good care and assuming responsibility, while at the same time respecting the patient's integrity, his/her right to self‐determination and information, and protecting human rights.^8^ Professional judgement on these issues is required, but to date the values, beliefs and previous experiences of patients with mental illness have not been explicitly included in structured evaluations in real medical practice.^17^

The aim of the present review is to assess the scope of the literature on the capacity of people with schizophrenia or bipolar disorder to make decisions about treatments in the management of their disease in real life. Building up evidence on the capacity of people with schizophrenia and bipolar disorder to make decisions about their illness is a first step on the way to a fuller consideration of their autonomy in usual medical practice.

## 2 METHODS

A systematic search of the literature was conducted observing the Preferred Reporting Items for Systematic reviews and Meta‐Analyses extension for Scoping Reviews (PRISMA‐ScR) Checklist^18^ to achieve the aim of the review. Free text searches and medical subject headings were combined to identify articles published in English and indexed in PubMed, Scopus, the *Cumulative Index to Nursing and Allied Health Literature (*CINAHL) and PsycInfo since the date of their indexation up to January 31, 2020. The search strategy is summarized in Appendix S1. Lists of references in the key papers retrieved were further checked to identify other relevant articles.

Inclusion criteria were any observational, real world study on schizophrenia, schizophrenic disorder, bipolar disorder, or psychosis individuals reporting a qualitative and/or quantitative assessment of patients' capacity to make treatment related decisions, including to consent and to make advance directives, and to express preferences for their psychiatric medications. Publications referring to multiple mental illnesses were included if the largest proportion of participants in the study were schizophrenia, bipolar disorder, or psychosis patients. The inclusion and exclusion criteria are shown in Table 1.

**Table 1:** Selection criteria

Inclusion criteria	Exclusion criteria
Topics Capacity to make decisions about treatment/s Capacity to consent to treatment Capacity to make advance directives regarding treatment of disease Disease Schizophrenia, schizoaffective disorders, bipolar disorder, psychosis Type of study Observational, real world evidence studies on decisional capacity Qualitative and/or quantitative assessment of decisional/preferences elicitation capacity Language of publication English Setting Any setting (inpatient, outpatient, forensic)	Animal, in vitro, or other types of pre‐clinical study Studies on dementia, Down syndrome, attention deficit‐ hyperactivity disorders, autism spectrum disorders, learning‐, sleep‐, eating‐hoarding‐, gambling‐ personality‐ or dissociative‐ disorders, Studies of decision making in presence of tumours of the central nervous system; on cognitive deficits that occur in the context of progressive chronic diseases (eg, multiple sclerosis, cardiovascular, respiratory, infection diseases) Newborn, infant, child, or adolescent studies Intellectual, developmental, and learning disability studies Validation tool studies Clinical practice guidelines Studies on professionals' and carers' decision making Studies on health‐ and social‐care services provision planning Studies on consent to research Studies on interventions to improve decision making capacity in mental disorder patients Studies on shared decision making Literature reviews Conceptual model studies

Potentially relevant abstracts were assessed by two expert reviewers Full text copies were requested of all papers initially considered suitable for inclusion in the review. Publications which were deemed pertinent after mutual agreement were reviewed and data were extracted. A third reviewer was involved in the selection process to resolve any disagreements. Data extraction was carried out by one researcher. A data extraction form that covered author, year of publication, country, study design, research tool, study objective, population and setting was designed and applied to summarize the key characteristics of publications (Table 2).

**Table 2:** Summary of studies reviewed on schizophrenia and bipolar disorder patients' capacity to make decisions about treatments and to consent to treatment

Author (year)	Country	Design	Research tool	Objective	Population: diagnosis, n	Setting
Bilanakis et al^19^	Greece	Cross‐sectional, semi‐structured interviews within 72 hours of admission	MacCAT‐T BPRS	Treatment decision‐making capacity	Schizophrenia, 21 Internal medicine, 78	Inpatients
Boettger et al^20^	Switzerland and United States	Cross‐sectional, retrospective, review of consultations for decisional capacity assessment	Descriptive statistics	Assessment of decisional capacity across a wide spectrum of medical and psychiatric disorders.	All patients, 336 Psychosis: stable, on antipsychotic medication, 22.6% Cognitive disorders: delirium and dementia, 42.6% Substance abuse disorder (active substance abuse prior to hospitalization: alcohol, opiates, and benzodiazepines; detoxification, and stable/dormant substance abuse: methadone maintenance), 41.3% Others	Inpatients
Brown et al^21^	United Kingdom	Cohort, retrospective assessment of case registreis	Mental Capacity Act 2005 criteria	Evaluation of the frequency mental capacity is assessed in psychiatric inpatients, whether the criteria for determining capacity set out in the Mental Capacity Act 2005 are used in practice, and whether this has increased with the introduction of the Mental Capacity Act 2005	Schizophrenia, 547 Schizoaffective and other psychotic disorders 268 Bipolar disorder, 232 Others	Inpatients
Cairns et al^22^	United Kingdom	Cross‐sectional, semi‐structured interviews 9 days of admission	MacCAT‐T BPRS BPCS MMSE SAI–E	Prevalence of psychiatric inpatients who lack capacity to make decisions about current treatment	Psychiatric patients, 112 (schizophrenia, 37; schizoaffective disorder, 11; other psychotic disorder, 14)	Inpatients
Capdevielle et al^23^	France	Cross‐sectional, semi‐structured interviews	MacCAT‐T SUMD PANSS	Competence to consent to treatment	Schizophrenia, 60	Outpatients
Curley et al^24^	Ireland	Cross‐sectional, semi‐structured interviews	MacCAT‐T	Mental capacity for treatment decisions, relationship between mental capacity (categorical) and various demographics and clinical variables	Psychiatry (schizophrenia or a related disorder and affective disorders) patients, 251	Inpatients
Curley et al^25^	Ireland	Cross‐sectional	MacCAT‐T	Mental capacity for treatment decisions, linear relationship between linear (as opposed to categorical) mental capacity and age.	Psychiatry (schizophrenia or a related disorder and affective disorders) patients, 215	Inpatients
Curley et al^26^	Ireland	Cross‐sectional	MacCAT‐T	Mental capacity for treatment decisions, comparison of assessments of mental capacity based on legal criteria with assessments based on clinical criteria	Psychiatry (schizophrenia or a related disorder and affective disorders) patients, 215	Inpatients
Dornan et al^27^	Ireland	Cohort, prospective, time one and time two structured interviews	MacCAT‐T	Changes of mental capacity to make treatment decisions over time	Schizophrenia patients, 37	Inpatients
Fernandez et al^28^	Ireland	Cohort, prospective, baseline, time one (6 weeks) and time two (12 weeks) structured interview	MacCAT‐T PANSS	Capacity to consent to treatment on admission, at 6‐ and 12‐weeks following treatment.	Psychosis, 56	Inpatients
Ganzini et al^29^	United Satates	Cross sectional, retrospective review of electronic medical records and data warehouse	Descriptive statistics	Examination of the characteristics of Veterans with schizophrenia admitted for nonpsychiatric hospitalization	Schizophrenia, 84	Inpatients
^30^	Australia	Cross‐sectional, semistructured interviews	MacCAT‐T PANSS	Competence to give informed consent to treatment	Acute psychosis, 110 (schizophrenia, 64; schizoaffective disorder, 25; bipolar disorder, 21)	Inpatients
Kennedy^31^	Ireland	Cross‐sectional, structured interviews	MacCAT‐T MacCAT‐FP PANSS GAF	Determination of whether giving extra information impairs the mental capacity to make decisions about treatment.	Psychosis, 88	Inpatients
Mandarelli et al^32^	Italy	Cross‐sectional, consecutive series, semi‐structured interviews	MacCAT‐T 24‐item BPRS MMSE RCPM	Differences in capacity to consent to psychiatric treatment	Involuntary/voluntary hospitalized acute mental disorder patients, 30 (schizophrenia/schizoaffective disorder, 18; bipolar disorder, 7; obsessive compulsive disorder, 1; psychotic disorder not otherwise specified, 3; brief psychotic disorder, 1)	Inpatients
^33^	Italy	Cross‐sectional, consecutive series, semi‐structured interviews	MacCAT‐T 24‐item BPRS MMSE	Decision‐making capacity to consent to psychiatric treatment	Schizophrenia spectrum disorder patients, 65 Bipolar disorder patients, 47	Inpatients
Maxmin and Cooper^34^	United Kingdom	Cross‐sectional, consecutive series, semistructured interviews	MacCAT‐T	Prevalence and predictors of mental capacity to make treatment and admission decisions in older psychiatric inpatients	Dementia, 40 Depression, 37 Psychotic disorder, 9 Mania, 10 Other, 3	Inpatients
Nystazaky (2018)	Greece	Retrospective, cross‐sectional, correlational, semi‐structured interviews.	MacCAT‐T BPRS	Decision making capacity on treatment with long acting injectable antipsychotic medication	Schizophrenia and schizoaffective disorder, 65	Outpatients
Owen et al^35^	United Kingdom	Cross‐sectional descriptive, semi‐structured interviews	MacCAT‐T	Prevalence of mental capacity to make decisions on treatment	Acute psychiatric patients, 350 (schizophrenia, 25%; schizoaffective disorder, 6%; psychotic episode, 22%; BPD, 12%)	Inpatients
Owen et al^36^	United Kingdom	Cross‐sectional, consecutive series, semi‐structured interviews (Secondary analysis)	MacCAT‐T SAI‐E	Associations of mental capacity for treatment decision making with variables clinicians are more familiar with, especially insight.	Acute psychiatric patients, 200	Inpatients
Owen et al^37^	United Kingdom	Cross‐sectional, consecutive series, semi‐structured interviews (Secondary analysis)	MacCAT‐T	Individuals' views on treatment decisions after regaining capacity	Acute psychiatric patients, 115	Inpatients
Owen et al^38^	United Kingdom	Cross‐sectional consecutive series, semi‐structured interviews	MacCAT‐T Mental capacity act	Associations of regaining capacity to make treatmentdecisions following inpatient psychiatric treatment	Acute psychiatric patients, 115 (subanalysis of schizophrenia and schizoaffective disorder patients)	Inpatients
Owen et al^39^	United Kingdom	Secondary analysis of two cross‐sectional studies, semi‐structured interviews	MacCAT‐T	Comparison of decision‐making capacity for treatment	Acute psychiatric patients, 125 Acute medical patients (nonpsychiatric), 164	Inpatients
Palmer et al^40^	United States	Cross‐sectional, semistructured interviews	MacCAT‐T HCAT PANSS BPRS DRS	Treatment decision‐making capacity	Psychosis patients, 16 (11 schizophrenia, 3 schizoaffectivedisorder, 1 bipolar disorder, 1 psychosis, not specified) Controls (healthy individuals), 40 (middle‐aged and older patients)	Outpatients
Palmer et al^41^	United States	Cross‐sectional, semi‐structured interviews	MacCAT‐T	Range, stability, and correlates of treatment‐related decisional capacity with aging	Schizophrenia and schizoaffective disorders, 59 (schizophrenia, 49; schizoaffective disorder, 10) Controls (healthy individuals), 38 (middle‐aged and older patients)	Outpatients
Raffard et al^42^	France	Cross‐sectional, semistructured interviews	MacCAT‐T BCIS	Relationship between capacity to consent to medication and cognitive biases	Schizophrenia patients, 60	Outpatients
Rutledge et al^43^	Ireland	Cross‐sectional, semistructured interviews	MacCAT‐T MacCAT‐FP PANSS GAF	Determination of whether tests of fitness to plead and capacity to consent are independent of each other and independent of mental state and global function in psychosis	Psychosis, 102	Inpatients
Skipworth et al^44^	Australia	Cross‐sectional	MacCAT‐T	Assessment of the capacity to consent in forensic patients at different stages of recovery and consideration of the implications of respecting their competent treatment decisions	Psychosis (forensic), 109	Outpatients and inpatients
Spencer et al (2018)	United Kingdom	Cross‐sectional, semi‐structured interviews	MacCAT‐T MacCAT‐CR	Differences between decision‐making capacity for treatment and research	Schizophrenia and related psychoses patients, 84	Inpatients
Vollmann et al^45^	Germany	Cross‐sectional, semistructured interviews	MacCAT‐T	Investigation of the competence of patients with dementia, depression and schizophrenia to make treatment decisions.	Dementia, 31 Depression, 35 Schizophrenia, 43	Inpatients
Wong et al^46^	China	Semi‐structured interviews	MacCAT‐T	Decision‐making abilities regarding maintenance treatment following hospital discharge after a psychotic relapse	Schizophrenia patients, 81	Inpatients

Abbreviations: BPCS, Burlington psychological and counselling services; BPRS, Brief Psychiatric Rating Scale; DRS, Depression Rating Scale; GAF, global assessment of functioning; HCAT, Hopkins competency assessment test; MacCAT‐CR, MacArthur Competence Assessment Tool for Clinical Research; MacCAT‐FP, MacArthur Competence Assessment Tool for Fitness to Plead; MacCAT‐T, MacArthur Competence Assessment Tool for Treatment; MMSE, Mini‐Mental State Examination; PANSS, Positive and Negative Syndrome Scale; RCPM, Raven's Coloured Progressive Matrices; SAI‐E, sexual arousability inventory‐expanded; SUMD, scale to assess unawareness of mental disorder.

The appraisal of publications was based on the Newcastle Ottawa Scale for observational studies, developed to assess their quality and risk of bias (Table 3).^47^ The Newcastle Ottawa Scale evaluates three quality parameters (selection, comparability, and outcome) divided across eight specific items. Each item on the scale is scored either 0 or 1, except for comparability, which can be adapted to the specific topic of interest and may score up to 2 points. Thus, the maximum score for each study is 9: studies with scores below five are considered to represent a high risk of bias. The Newcastle Ottawa Scale is one of the most used worldwide, above all for observational studies.^47^

**Table 3:** Newcastle‐Ottawa Scale: summary of the quality of the studies reviewed on decisional capacity regarding treatments and consent to treatment in schizophrenia or bipolar disorder persons (cohort studies)

Author (year)	Selection	Comparability	Outcome	Total (maximum score 9*)
Bilanakis et al^19^	***	**	**	******* (7)
Boettger et al^20^	***	**	***	********(8)
Brown et al^21^	***	**	***	********(8)
Cairns et al^22^	**	**	***	******* (7)
Capdevielle et al^23^	**	**	**	******(6)
Curley et al^24^	**	*	**	****(5)
Curley et al^25^	**	*	**	****(5)
Curley et al^26^	**	*	**	****(5)
Dornan et al^27^	***	*	***	*******(7)
Fernandez et al^28^	***	*	**	******(6)
Ganzini et al^29^	***	‐	**	****(5)
Howe et al^30^	***	*	*	*****(5)
Kennedy et al (2009)	***	‐	***	******(6)
Mandarelli et al^32^	***	**	**	*******(7)
Mandarelli et al^33^	**	**	**	******(6)
Maxmin and Cooper^34^	***	*	***	*******(7)
**Nystazaky (2018)	***	**	***	********(8)
Owen et al^35^	**	*	***	******(6)
Owen et al^36^	**	*	**	*****(5)
Owen et al^37^	***	**	***	********(8)
Owen et al^38^	***	*	**	******(6)
Owen et al^39^	***	**	***	********(8)
Palmer et al^40^	**	*	***	******(6)
Palmer et al^41^	**	**	***	*******(7)
Raffard et al^42^	***	**	***	********(8)
Rutledge et al^43^	***	‐	***	******(6)
Skipworth et al^44^	***	—	***	******(6)
Spencer et al (2018)	***	**	***	********(8)
Vollmann et al^45^	***	**	***	********(8)
Wong et al^46^	***	**	***	********(8)

*Note*: A study can be awarded a maximum of one star for each numbered item within the selection (five items) and outcome (two items) categories. A maximum of two stars can be given for comparability.

## 3 RESULTS

**Figure 1:**
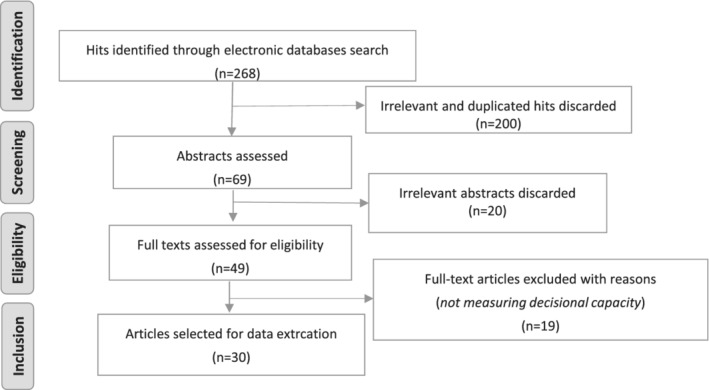
Flow diagram of the literature selection process

Searches identified a total of 268 hits. After reading titles and abstracts and removing irrelevant and duplicates, 49 potentially relevant papers were retained. Of these, 19 were excluded after assessing the full texts and 30 publications were finally reviewed for data extraction (Figure 1).

Most studies (23 out of 30) were carried out in European countries and assessed healthcare and treatment decisional capacity in the inpatients setting (24 out of 30 studies). Semistructured interviews based on the MacArthur Competence Assessment Tool for Treatment (MacCAT‐T) was the most frequently used method for assessing capacity (Table 2).

After assessing the characteristics and the nature of the evidence that emerged, publications were grouped into six main conceptual themes, as follows: (a) Insight as a determinant of decisional capacity; (b) Cognitive performance, and appreciation, as additional determinants of decisional capacity; (c) Treatment related decision‐making capacity maintenance despite partial impairments; (d) Retaining treatment related decision‐making capacity while in hospital; (e) Involuntary admission, or involuntary treatment, as drivers of decisional capacity impairments; (6) Regaining treatment related decision‐making capacity after impairments.

### 3.1 Insight is a key determinant of capacity to consent to treatment and to decide about treatment alternatives in patients with schizophrenia and related disorders

The studies conducted by Capdevielle et al^23^ and Raffard et al^42^ coincidently showed that insight (awareness of the disease) was a good indicator of capacity for deciding about treatments in outpatients with schizophrenia.^23^, ^42^ After studying 60 schizophrenic outpatients, negative correlations were found between the dimension “understanding” of the MacCAT‐T and the negative and the total Positive and Negative Syndrome Scale (PANSS) scores, and between the “appreciation,” “reasoning,” and “expressing a choice” MacCAT‐T dimensions and the Scale to Assess Unawareness of Mental Disorder (SUMD) dimensions. These findings imply that less symptomatic patients as well as those with a higher level of comprehension of the mental disorder and its consequences would have greater decisional capacity. An important correlation between the competence to consent to treatment and insight was reported in this population.^23^ Raffard et al^42^ explored the relationship between capacity to consent to medication and cognitive biases in 60 schizophrenia outpatients treated with antipsychotics for 1 month and found similar correlations. The authors of both publications shared the conclusion that higher levels of insight were associated with a greater appreciation of both the benefits and the risks of treatment, with a larger ability to make comparisons with alternative treatments and to express a choice between recommended treatments irrespective of the level of understanding. Furthermore, higher levels of objectivity, reflectiveness and openness to others' feedback were related to a greater ability to compare the prescribed treatment with alternatives, to discuss the consequences of treatment alternatives, and to evaluate their impact on everyday life.^23^, ^42^

### 3.2 Cognitive performance, and appreciation, further determines decisional capacity in psychotic disorder patients

Palmer et al^41^ examined whether the cognitive changes associated with normal aging had a negative impact on decision‐making processes in patients with psychotic disorders and without dementia. It turned out that schizophrenia or schizoaffective disorder patients were as good as non‐schizophrenia individuals in reasoning and expressing of a choice, even when age was added as a covariate. Also, the patients' level of decisional capacity was stable during the 1‐month follow‐up. Schizophrenia patients scored lower only on understanding treatment‐related disclosures, but with high interpersonal variability. Thus, patients' level of capacity was not associated with age, or with the severity of the psychopathology, but it was strongly associated with cognitive test performance.^41^ Similarly, other studies reported that as many as 52.5% and 38.4% of older psychiatric inpatients had capacity to make admission and treatment related decisions, respectively, and that capacity was associated with not having dementia, and with higher levels of insight and cognition.^34^

Howe et al^30^ investigated the association between the capacity to give consent to treatment, specific symptomatology, and diagnosis in 110 patients with acute schizophrenia, schizoaffective disorder and bipolar disorder admitted into hospital, all at an early stage of treatment for acute psychosis. Lacking capacity to consent to treatment was related to more severe cognitive dysfunction, conceptual disorganization, and poor attention. Noticeable, the authors found that decisional capacity was independent of the psychotic disorder being diagnosed.^30^

Findings from other studies further substantiated the notion of adequate cognitive performance for preserving healthcare decision‐making capacity. It was documented that cognitive disorders (delirium, dementia) affected the ability to make healthcare decisions the most (54.1%) compared to other psychiatric comorbidities and interfered more commonly with decisional capacity than psychotic disorders (25%).^20^ Among the patients able to express a choice about treatments in the psychiatric hospital where psychotic and severe affective disorders predominated (37%), the ability to appreciate the relevance of the information (appreciation) was a reliable indicator of their decision‐making capacity.^39^ Vollman et al^45^ also found that schizophrenia patients generally appreciated the treatment benefit despite a reduced appreciation of the mental disorder, and were more often classified as impaired by the MacCAT‐T subscales than by clinical assessment (53.5% vs 18.4%) depending on the cut‐offs applied for establishing incompetence.^45^

### 3.3 Treatment related decision‐making capacity is preserved despite partial impairments while in the community or in hospital

Nystazaki et al (2018) found that more than 70% of 65 outpatients diagnosed with schizophrenia and schizoaffective disorder understood the relevance of the information related to the use of long acting injectable antipsychotic medications. However, understanding of the information on their medical condition was relatively poor (as assessed with the MacCAT‐T subscales). Despite such partial impairments, the authors concluded that none of the patients *completely* lacked decision‐making capacity. Very much in line with these findings, Skipworth et al^44^ reported that the majority of forensic patients (67.6% of a sample of 109) had treatment related capacity both in hospital and in community settings, and very few of those patients with capacity refused psychiatric treatment.^44^ Earlier, Palmer et al^40^ had demonstrated that understanding improved significantly over time and with repeated presentations of the information in a smaller population of schizophrenia and schizoaffective disorder patients^40^ while Raffard et al^42^ suggested that cognitive therapy, by enhancing patients' self‐reflectiveness and considering alternative explanations, could lead to better capacity to consent to treatment in schizophrenia.^42^ On the other hand, Kennedy et al^31^ found that the amount of information to be disclosed to psychosis patients' needs to be balanced. Giving too much extra information when obtaining consent for treatment for a psychotic illness may lead to a decline in the patients' ability to make a choice. Up to 15% of psychotic patients may become unable to decide about treatment options, particularly those with low or intermediate decisional capacity scores.^31^

### 3.4 Schizophrenia and bipolar disorder patients retain capacity for treatment related decision making after hospital admission

Spencer et al (2018) found that 31% (95% CI: 21%‐43%) of 84 unwell psychiatric inpatients admitted for the assessment and/or treatment of schizophrenia and related psychoses had capacity to make decisions about treatments, although different symptoms, such as delusions and hallucinations had different effects on decision‐making abilities. Ganzini et al^29^ reported that 42% of veterans with schizophrenia admitted for nonpsychiatric hospitalizations, such as infection, cardiac disease or altered mental status that required assessing the psychotropic medication lacked decision‐making capacity mostly secondary to delirium; 58% of these patients retained decision‐making capacity while in hospital.^29^

Cairns et al^22^ studied the capacity to make treatment decisions soon after admission to hospital in a total of 112 schizophrenia, schizoaffective disorder, and other psychotic disorder patients. More than half (56.2%) remained capable of making treatment‐related decisions, and decisional capacity was mostly present (90.5%) among those who had been voluntarily admitted. Bilanakis et al^19^ found that the capacity for decision making was compromised during the first 72 hours of admission in 21 patients with schizophrenia of whom 62% had been involuntarily admitted into a psychiatric ward in a general hospital. In both studies, decisional capacity was related to positive and negative psychiatric symptomatology while the phase of the disorder (mania, hypomania, delusional) temporarily and variably impaired decisional capacity.^19^, ^22^

Likewise, a study conducted in Ireland which included both voluntarily and involuntarily admitted individuals with any mental disorder found that 1.9% of participants lacked mental capacity for treatment decisions; 50.7% had partial mental capacity; and 47.4% had full mental capacity.^24^ With respect to the ability to understand information about diagnosis and treatment, 10.7% of participants lacked this ability; 38.6% had partial ability; and 50.7% had full ability. Greater mental capacity was significantly associated with voluntary admission status, Irish ethnicity, being in employment and of younger age. The authors also found that voluntary admission status, being employed, having a primary diagnosis other than schizophrenia or a related disorder, and younger age accounted together for only 44.4% of the variance in mental capacity implying that other unexplored factors contributed to decisional capacity.^25^ The clinical and legal criteria applied to assess mental capacity following the Ireland's Assisted Decision‐Making (Capacity) Act 2015 closely correlated. Thus, patients who lacked mental capacity according to the legislation scored significantly lower on all subscales of the MacCAT‐T than patients who had mental capacity.^26^

### 3.5 Involuntary admission, or involuntary treatment, compromises decisional capacity in schizophrenia and bipolar disorder patients

Mandarelli et al^32^ compared the capacity ratings of patients treated voluntarily and involuntarily in a psychiatric acute care unit to consent to psychiatric treatment. Patients treated voluntarily scored considerably better than those treated involuntarily in all MacCAT‐T subscales and were more able than those admitted involuntarily to understand, appreciate, and reason about their own clinical condition, the risks, and benefits of treatment, and to express a clear treatment choice.^32^ A subsequent study by the same authors found that 22% (n = 29) of 131 patients with an acute psychotic episode involuntarily hospitalized and treated also had high treatment decision‐making capacity, defined as scoring above 75% of the maxima in all four MacCAT‐T subscales.^33^ Likewise, a study by Brown et al^21^ reported that 67.1% (95% CI: 63.1‐71.0) of schizophrenia; 60.8% (95% CI: 54.9‐66.7) of schizoaffective/other psychotic and 69.0% (95% CI: 63.0‐75.0) of bipolar disorder patients were assessed to lack capacity at psychiatric admission according to the Mental Capacity Act 2005 criteria. About 30% to 40% of these patients remained capable of decision making. Incapacity was more frequent among those admitted involuntarily.^21^ Similarly, Rutledge et al^43^ reported that compulsory detained patients with psychosis and incapable of making a treatment choice scored significantly worse in all rating scales, including the MacCAT‐T, MacCAT‐FP, PANSS, and the Global Assessment of Functioning (GAF).^43^

### 3.6 Schizophrenia and bipolar disorder patients regain capacity for treatment related decision making after impairments while in hospital

In the United Kingdom, Owen and collaborators conducted a series of studies to assess decision‐making capacity in psychiatric inpatients admitted to acute psychiatric wards. They found that the capacity to make treatment related decisions was compromised in up to 60% (95% CI: 55%‐65%) in people admitted involuntarily suggesting that 40% of these patients retained decisional capacity despite the stressful situation they were experiencing.^35^ Maniac episodes among bipolar disorder patients and the burden of psychopathological symptoms overall were strongly associated with incapacity.^36^ Among those labeled as incapable at admission, 83% regained capacity to make treatment decisions after 1 month of treatment^37^ and were able to retrospectively approve the decisions made by psychiatrists on their behalf while impaired.^38^ Insight was found to be the best discriminator of the status of capacity among psychotic inpatients.^35^, ^36^, ^38^

Dornan et al^27^ were also interested in quantifying the relationship of decisional capacity to time. They used competence assessment tools, and rating scales for symptoms and global function in 37 inpatients, all with psychosis in a secure psychiatric hospital. Patients were interviewed twice a mean of 323 days apart (median 176 days; range 17‐1221 days). The number judged by treating psychiatrists to lack capacity either to make a treatment choice or to plead in court fell from 35% to 8% demonstrating that there was an improvement in capacity scores with time. There also was a strong relationship between the clinicians' assessment of capacity and structured rating scales.^27^

In 56 patients with psychosis, Fernandez et al^28^ studied their capacity to consent to treatment after involuntary hospital admission and at 6‐ and 12‐weeks following treatment. At the time of admission, 62.5% had decisional capacity and 37.5% of participants lacked it; this latter figure dropped to 17.9% at 6 weeks and to 5.4% at 12 weeks of treatment, showing that decision‐making capacity improved over 12 weeks of treatment.^28^

Wong et al^46^ interviewed 81 schizophrenia patients before their discharge from hospital after a psychotic relapse to examine their decisions on whether to take maintenance treatment. The authors found that 79% of participants had scores above 4 on understanding, 74.1% above 6 on reasoning and 82.7% above 3 on the appreciation subscales of the MacCAT‐T, indicating that most patients had the ability to make decisions with regard to following the treatment recommended at discharge from hospital.^46^

### 3.7 Quality appraisal of publications

According to the Newcastle‐Ottawa Scale criteria for assessing the methodological quality of studies, most of the publications reviewed obtained five to eight stars out of nine and were therefore judged to be of high quality.^47^

## 4 DISCUSSION

This review provides data on the capacity of schizophrenia and bipolar disorder patients for making autonomous decisions regarding the treatment of their disease. The proportion of participants in the studies reviewed who had capacity for making appropriate treatment decisions went beyond 70% among outpatients as indicated by their competent understanding of treatment options, and decisional capacity was satisfactorily regained following treatment among hospital inpatients. Similar to patients with a nonpsychiatric condition like diabetes, obesity or old age, schizophrenia and bipolar disorder persons in the community were able to take part in assessments in a way that reflected their own choices.^48^ Elements such as cognitive capacity, physical functioning, and level of education all contribute to decision‐making performance among psychiatric and nonpsychiatric patients, even after adjusting for diagnosis^49^.^50^, ^51^ These findings support the overriding principle that schizophrenia or bipolar disorder patients must be assumed to have capacity unless established otherwise, and that they should not to be treated as unable to make a decision unless all practicable steps to help them to do so have been taken without success.^52^

More than 30% of inpatients with severe symptoms and long‐standing disease was able to make treatment decisions soon after hospital admission in the publications reviewed. Although in the moment of admission, capacity impairments could limit autonomous critical decisions such as voluntary or involuntary admission or changes in the treatment plan, by the time of hospital discharge, the majority of schizophrenia or bipolar disorder patients had recovered decision‐making capacity. In this sense, there is evidence that substantiates the notion that even symptomatic bipolar disorder or schizophrenia patients can be capable of distinguishing, describing, and evaluating their own health states.^53^, ^54^

The review also shows that, in mental health research, the capacity for decision making is usually assessed according to the four traditional criteria of understanding, appreciation, reasoning, and expressing a choice^9^ and that each of these elements contributes in different ways to the person's decisional capacity. The effects of impairments in schizophrenia and bipolar disorder on patients' decision‐making and functional capacity may vary in intensity depending on the individual, the phase of the illness, the prevailing psychotic symptoms, cognitive function, the moment in time, and the type of decision to be made.^55^

The understanding of the disease‐ and treatment‐related information is commonly impaired in schizophrenia and bipolar disorder patients, but this does not mean that these individuals are incapable of making their own decisions or that they are unable to adequately perform treatment‐related tasks.^56^

The level of understanding can be easily improved by adopting measures such as repeating and redisclosing the missed information or using enhanced information procedures.^57^ Research into functional capacity shows that schizophrenia patients can normally manage medications and keep prescription refills over time.^58^ These findings reinforce the notion that capacity is a complex, dynamic, and multifactorial neurocognitive concept that should be properly assessed and re‐assessed by the clinician familiar with the patient or with the nature of the disease. They also show that, when present, the loss of the capacity for decision making is temporary and the ability recovers over time in the vast majority of patients with schizophrenia or bipolar disorder.^59^ Therefore, capacity assessments should primarily be undertaken not to judge whether people are capable or not to decide autonomously, but rather to assess what kind of support people with decision‐making impairments need in order to be involved in decision making, and thus to promote their autonomy.^60^

The psychiatrist's clinical judgment is fundamental in assessing the decision‐making capacity of mentally disordered patients^21^ .^22^ The currently available scales are very limited and are poorly suited for evaluating this capacity. In this context, a specific assessment of capacity should be conducted together with the standard routine medical and psychopathological evaluation in any patient in a crisis episode and when preparing a shared contract or a joint care plan. Healthcare practitioners should remember that the limitation of capacity is temporary, and a reassessment should therefore be performed within a reasonable period of time.

The limitations of the review are related to the type of the studies included and the fact that only publications in English existing in four electronic sources were accepted. Although the review is comprehensive, relevant studies published in other languages and indexed in other databases may have not been identified. Furthermore, papers on interventions for improving decisional capacity were excluded which can miss some potential eligible studies that might report the capacity of schizophrenia or bipolar disorder individuals before the intervention. Most reviewed research was conducted in small populations from individual services making results less representative of all patterns of care at regional or country level. The internal consistency of the studies may be low due to their observational nature, although they are highly reflective of everyday medical practice with a high external validity.

Overall, common methods and more robust designs are needed to advance knowledge in this highly relevant topic. Future studies should account for generalisability and allow international comparisons taking into account differing requirements of capacity for different healthcare decisions, such as crisis management, hospitalisation, containment, and long‐term treatment plans. Also, cultural variations, the disparity across mental health conditions and diversity among legal frameworks should be considered in future research. Similarly, more research is needed in relation to the assessment of patients' capacity in the emergency room, the comparative analysis of the psychometric properties and usability of screening questionnaires for the assessment of capacity in different contexts and for different purposes, and guidelines for the assessment of capacity in severe mental disorders beyond the extended use of MacCAT‐T. and in medical conditions.^61^

To conclude, the studies assessed reflect that knowledge on the decisional capacity of schizophrenia or bipolar disorder patients come mostly from the hospital environment in which more severely ill individuals are cared for. Less research has been conducted in individuals while in the community. Despite the greater burden of illness in the studied populations, the evidence shows that schizophrenia and bipolar disorder individuals most often have capacity to make decisions about their medical treatment, and that the proportion of individuals with no treatment decisional capacity is actually very small. Therefore, the majority of patients with schizophrenia or bipolar disorder are capable of treatment related decision making and should be involved in decisions about the care of their health.

## FUNDING

Ferrer funded the development of the study and the writing of the manuscript with an unrestricted grant.

## CONFLICT OF INTEREST

The authors declare that they have no conflict of interest.

## AUTHOR CONTRIBUTIONS

Conceptualization: Alfredo Calcedo‐Barba, Enric Vincens Pons, Silvia Paz

Formal Analysis: Alfredo Calcedo‐Barba, Enric Vincens Pons, Luis Salvador‐Carulla, Bruno Paccardi, Silvia Paz

Funding Acquisition: Alfredo Calcedo‐Barba, Enric Vincens Pons

Writing – review and editing: Silvia Paz, Alfredo Calcedo‐Barba, Enric Vincens Pons, Luis Salvador‐Carulla, Bruno Paccardi, Thomas Messer, Scott L. Zeller

Writing – original draft: Silvia Paz

All authors read and approved the final manuscript.

Alfredo Calcedo‐Barba had full access to all of the data in this study and takes complete responsibility for the integrity of the data and the accuracy of the data analysis.

## TRANSPARENCY STATEMENT

Alfredo Calcedo‐Barba affirms that this manuscriptis an honest, accurate, and transparent account of the study being reported; that no important aspects of the study have been omitted; and that any discrepancies from the study as planned (and, if relevant, registered) have been explained.

## Supporting information


**Appendix S1**: Supporting Information

## Data Availability

The data that support the findings of this study are available from the corresponding author upon reasonable request.

## References

[1] Entwistle VA , Carter SM , Cribb A , McCaffery K . Supporting patient autonomy: the importance of clinician‐patient relationships. J Gen Intern Med. 2010;25(7):741‐745. 10.1007/s11606-010-1292-2. 20213206 PMC2881979

[2] Coggon J , Miola J . Autonomy, liberty and medical decision‐making. Camb Law J. 2011;70(3):523‐547. 10.1017/S0008197311000845. 23293377 PMC3535760

[3] Varelius J . The value of autonomy in medical ethics. Med Health Care Philos. 2006;9(3):377‐388. 10.1007/s11019-006-9000-z. 17033883 PMC2780686

[4] Eassey D , Reddel HK , Ryan K , Smith L . The impact of severe asthma on patients' autonomy: a qualitative study. Health Expect. 2019;22(3):528‐536. 10.1111/hex.12879. 30900374 PMC6543152

[5] Proot I et al. Stroke patients' needs and experiences regarding autonomy at discharge from nursing home. Patient Educ Couns. 2000;41(3):275‐283. 11042430 10.1016/s0738-3991(99)00113-5

[6] Coggon J . Mental capacity law, autonomy, and best interests: an argument for conceptual and practical clarity in the court of protection. Med Law Rev. 2016;24(3):396‐414. 10.1093/medlaw/fww034. 28007810 PMC5178324

[7] Slade M , Amering M , Farkas M , et al. Uses and abuses of recovery: implementing recovery‐oriented practices in mental health systems. World Psychiatry. 2014;13(1):12‐20. 10.1002/wps.20084. 24497237 PMC3918008

[8] Pelto‐Piri V , Engström K , Engström I . Paternalism, autonomy and reciprocity: ethical perspectives in encounters with patients in psychiatric in‐patient care. BMC Med Ethics. 2013;14(1). Published 2013 Dec 6. 10.1186/1472-6939-14-49. PMC4029406 24314345

[9] Hermann H et al. Emotion and value in the evaluation of medical decision‐making capacity: a narrative review of arguments. Front Psychol. 2016;7:1‐10. 10.3389/fpsyg.2016.00765. 27303329 PMC4880567

[10] Lai JM , Karlawish J . Assessing the capacity to make everyday decisions: a guide for clinicians and an agenda for future research. Am J Geriatr Psychiatry. 2007;15(2):101‐111. 10.1097/01.JGP.0000239246.10056.2e. 17272730

[11] Cáceda R , Nemeroff CB , Harvey PD . Toward an understanding of decision making in severe mental illness. J Neuropsychiatry Clin Neurosci. 2014;26(3):196‐213. 10.1176/appi.neuropsych.12110268. 24599051

[12] Wagner LC Torres‐González F, Geidel AR, King MB. Existential questions in schizophrenia: perception of patients and caregivers. Rev Saude Publica. 2011;45(2):401‐408. https://doi:10.1590/s0034-89102011000200019 21412576 10.1590/s0034-89102011000200019

[13] Hajda M , Prasko J , Latalova K , et al. Unmet needs of patients with bipolar disorder. Neuropsychiatr Dis Treat. 2016;12:1561‐1570. 27445475 10.2147/NDT.S105728 PMC4928671

[14] Tibaldi, G. , Salvador‐Carulla, L. and García‐Gutierrez, J. (2011) ‘From treatment adherence to advanced shared decision making: new professional strategies and attitudes in mental health care.’, Curr Clin Pharmacol. 6(2):91–99. 10.2174/157488411796151101. 21592062

[15] Aimola L , Gordon‐Brown J , Etherington A , Zalewska K , Cooper S , Crawford MJ . Patient‐reported experience and quality of care for people with schizophrenia. BMC Psychiatry. 2019;19(1):17 10.1186/s12888-018-1998-y. 30626355 PMC6327578

[16] Dempsey T , DeMartino E . How should clinicians navigate decision making for unrepresented patients? AMA J Ethics. 2019;21(7):E559‐E565. 10.1001/amajethics.2019.559. 31333170

[17] Karel MJ , Gurrera RJ , Hicken B , Moye J . Reasoning in the capacity to make medical decisions: the consideration of values. J Clin Ethics. 2010;21(1):58‐71. 20465077 PMC3034382

[18] Tricco AC , Lillie E , Zarin W , et al. PRISMA extension for scoping reviews (PRISMA‐ScR): checklist and explanation. Ann Intern Med. 2018;169(7):467‐473. 10.7326/M18-0850. 30178033

[19] Bilanakis N , Peritogiannis V , Vratsista A . Treatment decision‐making capacity in hospitalized patients with schizophrenia. Psychiatriki. 2017;28(1):37‐45. 10.22365/jpsych.2017.281.37. 28541237

[20] Boettger S , Bergman M , Jenewein J , Boettger S . Assessment of decisional capacity: prevalence of medical illness and psychiatric comorbidities. Palliat Support Care. 2015;13(5):1275‐1281. 10.1017/S1478951514001266. 25355466

[21] Brown PF , Tulloch AD , Mackenzie C , Owen GS , Szmukler G , Hotopf M . Assessments of mental capacity in psychiatric inpatients: a retrospective cohort study. BMC Psychiatry. 2013;13 Published 2013 Apr 15. 10.1186/1471-244X-13-115. PMC3643852 23586975

[22] Cairns R , Maddock C , Buchanan A , et al. Prevalence and predictors of mental incapacity in psychiatric in‐patients. Br J Psychiatry. 2005;187(04):379‐385. 10.1192/bjp.187.4.379. 16199799

[23] Capdevielle D , Raffard S , Bayard S , et al. Competence to consent and insight in schizophrenia: is there an association? A pilot study. Schizophr Res. 2009;108(1–3):272‐279. 10.1016/j.schres.2008.12.014. 19162443

[24] Curley A , Murphy R , Fleming S , et al. Age, psychiatry admission status and linear mental capacity for treatment decisions. Int J Law Psychiatry. 2019a;66:101469 10.1016/j.ijlp.2019.101469. 31706384

[25] Curley A , Murphy R , Plunkett R , et al. Categorical mental capacity for treatment decisions among psychiatry inpatients in Ireland. Int J Law Psychiatry. 2019b;64:53‐59. 10.1016/j.ijlp.2019.02.001. 31122640

[26] Curley A , Murphy R , Plunkett R , et al. Concordance of mental capacity assessments based on legal and clinical criteria: a cross‐sectional study of psychiatry inpatients. Psychiatry Res. 2019c;276:160‐166. 10.1016/j.psychres.2019.05.015. 31096146

[27] Dornan J , Kennedy M , Garland J , Rutledge E , Kennedy HG . Functional mental capacity, treatment as usual and time: magnitude of change in secure hospital patients with major mental illness psychiatry. BMC Res Notes BioMed Central. 2015;8(1):1‐9. 10.1186/s13104-015-1547-4. PMC4606547 26467781

[28] Fernandez C , Kennedy HG , Kennedy M . The recovery of factors associated with decision‐making capacity in individuals with psychosis. BJPsych Open. 2017;3(3):113‐119. 10.1192/bjpo.bp.116.004226. 28507770 PMC5415675

[29] Ganzini L , Mansoor D , Socherman R , Duckart J . Delirium and decisional incapacity in veterans with schizophrenia and medical illness. Gen Hosp Psychiatry. 2012;34(5):506‐509. 22632926 10.1016/j.genhosppsych.2012.04.003

[30] Howe V , Foister K , Jenkins K , Skene L , Copolov D , Keks N . Competence to give informed consent in acute psychosis is associated with symptoms rather than diagnosis. Schizophr Res. 2005;77(2–3):211‐214. 10.1016/j.schres.2005.03.005. 16085206

[31] Kennedy M et al. Extra information about treatment is too much for the patient with psychosis. Int J Law Psychiatry. 2009;32(6):369‐376. 10.1016/j.ijlp.2009.09.006. 19793614

[32] Mandarelli G , Tarsitani L , Parmigiani G , et al. Mental capacity in patients involuntarily or voluntarily receiving psychiatric treatment for an acute mental disorder. J Forensic Sci. 2014;59(4):1002‐1007. 10.1111/1556-4029.12420. 24502678

[33] Mandarelli G , Carabellese F , Parmigiani G , et al. Treatment decision‐making capacity in non‐consensual psychiatric treatment: a multicentre study. Epidemiol Psychiatr Sci. 2018;27(5):492‐499. 10.1017/S2045796017000063. 28274298 PMC6999012

[34] Maxmin K , Cooper C . Mental capacity to consent to treatment and admission decisions in older adult psychiatric inpatients. Int J Geriatr Psychiatry. 2009;24:1367‐1375. 10.1002/gps. 19378346

[35] Owen GS et al. Mental capacity to make decisions on treatment in people admitted to psychiatric hospitals: cross sectional study. BMJ. 2008;337 Published 2008 Jun 30. 10.1136/bmj.39580.546597.BE. PMC2443596 18595931

[36] Owen G et al. Retrospective views of psychiatric in‐patients regaining mental capacity. Br J Psychiatry. 2009a;195(5):403‐407. 10.1192/bjp.bp.109.065151. 19880929 PMC2806572

[37] Owen GS , David AS , Richardson G , Szmukler G , Hayward P , Hotopf M . Mental capacity, diagnosis and insight in psychiatric in‐patients: a cross‐sectional study. Psychol Med. 2009b;39(8):1389‐1398. 10.1017/S0033291708004637. 18940026 PMC7611628

[38] Owen GS , Ster IC , David AS , et al. Regaining mental capacity for treatment decisions following psychiatric admission: a clinico‐ethical study. Psychol Med. 2011;41(1):119‐128. 10.1017/S0033291710000383. 20346192 PMC7611689

[39] Owen GS , Szmukler G , Richardson G , et al. Decision‐making capacity for treatment in psychiatric and medical inpatients: cross‐sectional, comparative study. Br J Psychiatry. 2013;203(6):461‐467. 10.1192/bjp.bp.112.123976. 23969482 PMC3844898

[40] Palmer BW , Nayak GV , Dunn LB , Appelbaum PS , Jeste DV . Treatment‐related decision‐making capacity in middle‐aged and older patients with psychosis: a preliminary study using the MacCAT‐T and HCAT. Am J Geriatr Psychiatry. 2002;10(2):207‐211. 10.1097/00019442-200203000-00012. 11925282

[41] Palmer B et al. Correlates of treatment‐related decision‐making capacity among middle‐aged and older patients with schizophrenia. Arch Gen Psychiatry. 2004;61(3):230‐236. 14993110 10.1001/archpsyc.61.3.230

[42] Raffard S , Fond G , Brittner M , et al. Cognitive insight as an indicator of competence to consent to treatment in schizophrenia. Schizophr Res. 2013;144(1–3):118‐121. 10.1016/j.schres.2012.12.011. 23313358

[43] Rutledge E , Kennedy M , O'Neill H , Kennedy HG . Functional mental capacity is not independnet of the severity of psychosis. Int J Law Psychiatry. 2008;31(1):9‐18. 18164385 10.1016/j.ijlp.2007.11.002

[44] Skipworth J , Dawson J , Ellis P . Capacity of forensic pateints to consent to treatment. Aust New Zealand J Psychiatry. 2013;47(5):443‐450. 23172655 10.1177/0004867412468495

[45] Vollmann J et al. Competence of mentally ill patients: a comparative empirical study. Psychol Med. 2003;33(8):1463‐1471. 10.1017/S0033291703008389. 14672255

[46] Wong J , Cheung E , Chen E . Decision‐making capacity of inpatients with schizophrenia in Hong Kong. J Nerv Ment Dis. 2005;193(5):316‐322. 15870615 10.1097/01.nmd.0000161685.54077.e4

[47] Luchini C , Stubbs B , Solmi M , Veronese N . Assessing the quality of studies in meta‐analyses: advantages and limitations of the Newcastle Ottawa Scale. World J Meta‐Anal. 2017;5(4):80 10.13105/wjma.v5.i4.80.

[48] Bridges J et al. Can patients diagnosed with schizophrenia complete choice‐based conjoint analysis tasks? Patient. 2011;4(4):267‐275. http://ovidsp.ovid.com/ovidweb.cgi?T=JS&PAGE=reference&D=emed10&NEWS=N&AN=2011571296. 21995832 10.2165/11589190-000000000-00000

[49] Candilis P et al. A direct comparison of research decision‐making capacity: schizophrenia/schizoaffective, medically ill, and non‐ill subjects. Schizophr Res. 2008;99(1–2):350‐358. 10.1371/journal.pone.0178059. 18164593 PMC2486336

[50] Restivo MR , McKinnon MC , Frey BN , Hall GB , Syed W , Taylor VH . The impact of obesity on neuropsychological functioning in adults with and without major depressive disorder. PLoS ONE. 2017;12(5):1‐15. 10.1371/journal.pone.0176898. PMC5419516 28475603

[51] Luck‐Sikorski C , Stein J , Heilmann K , et al. Treatment preferences for depression in the elderly. Int Psychogeriatr. 2017;29(3):389‐398. 27890036 10.1017/S1041610216001885

[52] NICE . (2018) Decision‐making and mental capacity ‐ NICE guideline. https://www.nice.org.uk/guidance/ng108/resources/decisionmaking‐and‐mental‐capacity‐pdf‐66141544670917.

[53] Banihashemian M , Rashidian A , Gholamian F , Parsaeian M , Moradi N , Amini H . Health state utilities for patient's current health from bipolar type I disorder. J Ment Health Policy Econ. 2018;21(1):3‐10. 29643263

[54] Voruganti L et al. Assessing health utilities in schizophrenia. A feasibility study. Pharmacoeconomics. 2000;17(3):273‐286. 10.2165/00019053-200017030-00005. 10947302

[55] Rajji T , Miranda D , Mulsant B . Cognition, function, and disability in patients with schizophrenia: a review of longitudinal studies. Can J Psychiatry. 2014;59(1):13‐17. http://www.pubmedcentral.nih.gov/articlerender.fcgi?artid=4079219&tool=pmcentrez&rendertype=abstract. 24444319 10.1177/070674371405900104 PMC4079219

[56] Sugawara N , Yasui‐Furukori N , Sumiyoshi T . Competence to consent and its relationship with cognitive function in patients with schizophrenia. Front Psych. 2019;10(April):1‐5. 10.3389/fpsyt.2019.00195. PMC6474312 31031653

[57] Palmer B et al. Decisional capacity to consent to research among patients with bipolar disorder: comparison with schizophrenia patients and healthy subjects. J Clin Psychiatry. 2007;68(5):689‐696. 17503977 10.4088/jcp.v68n0505

[58] Heinrichs R et al. Predictors of medication competence in schizophrenia patients. Psychiatry Res. 2008;157(1–3):47‐52. 17897721 10.1016/j.psychres.2007.02.015

[59] Ganzini L et al. Ten myths about decision‐making capacity. J Am Med Dir Assoc. 2004;5(4):263‐267. 15228638 10.1097/01.JAM.0000129821.34622.A2

[60] Peisah C , Sorinmade OA , Mitchell L , Hertogh CMPM . Decisional capacity: toward an inclusionary approach. Int Psychogeriatr. 2013;25(10):1571‐1579. 10.1017/S1041610213001014. 23809025 PMC3750821

[61] Scholz B , Bocking J , Platania‐Phung C , Banfield M , Happell B . “Not an afterthought”: power imbalances in systemic partnerships between health service providers and consumers in a hospital setting. Health Policy. 2018;122(8):922‐928. 10.1016/j.healthpol.2018.06.007. 30017107

[62] Nystazaki M , Pikouli K , Tsapakis EM , Karanikola M , Ploumpidis D , Alevizopoulos G . Decision‐making Capacity for Treatment of Psychotic Patients on Long Acting Injectable Antipsychotic Treatment. Arch Psychiatr Nurs. 2018;32(2):300‐304. 29579528 10.1016/j.apnu.2017.11.019

