# Supplementary material for: The capacity of schizophrenia and bipolar disorder individuals to make autonomous decisions about pharmacological treatments for their illness in real life: A scoping review
Source: Health Sci Rep. 2020 Aug 9;3(3):e179. doi: 10.1002/hsr2.179 (PMC7415958; doi:10.1002/hsr2.179)
Supplement: Supplementary file 1 — Appendix S1: Supporting Information [file HSR2-3-e179-s001.docx]

Appendix 1

Search strategy

|  | **Databases** | **CINAHL, hits (n)** | **PubMed, hits (n)** | **PsycInfo, hits (n)** | **Scopus, hits (n)** |
| --- | --- | --- | --- | --- | --- |
| # | **Search terms, strategy** |  |  |  |  |
|  | Limit #12 #15 #20 to English language, Journal article, Humans |  |  |  |  |
| 1 | schizophrenia |  |  |  |  |
| 2 | psychosis |  |  |  |  |
| 3 | psychoses |  |  |  |  |
| 4 | psychotic disorder |  |  |  |  |
| 5 | schizophrenic disorder |  |  |  |  |
| 6 | bipolar disorder |  |  |  |  |
| 7 | decision making capacity |  |  |  |  |
| 8 | treatment |  |  |  |  |
| 9 | intervention |  |  |  |  |
| 10 | therapy |  |  |  |  |
| 11 | medication |  |  |  |  |
| **12** | **#1 OR #2 OR #3 OR #4 OR #5 OR #6 AND #7 AND #8 OR #9 OR #10 OR #11** | **18** | **78** | **64** | **108** |
| **Total hits per database (all search strategies)** | | **18** | **78** | **64** | **108** |
| **Total hits** | | **268** | | | |
